# Probabilistic Assessment of Fracture Toughness of Epoxy Resin EPOLAM 2025 Including the Notch Radii Effect

**DOI:** 10.3390/polym13111857

**Published:** 2021-06-03

**Authors:** Adrián Álvarez-Vázquez, Miguel Muñiz-Calvente, Pelayo Fernández Fernández, Alfonso Fernández-Canteli, María Jesús Lamela-Rey, José María Pintado

**Affiliations:** 1 Department of Construction and Manufacturing Engineering, University of Oviedo, 33203 Gijón, Spain; munizcmiguel@uniovi.es (M.M.-C.); fernandezpelayo@uniovi.es (P.F.F.); afc@uniovi.es (A.F.-C.); mjesuslr@uniovi.es (M.J.L.-R.); 2 National Institute for Aerospace Technology (INTA), 28850 Madrid, Spain; jmpintado@coiae.com

**Keywords:** notched components, failure probabilistic prediction, theory of critical distances

## Abstract

Many design scenarios of components made of polymer materials are concerned with notches as representative constructive details. The failure hazard assessment of these components using models based on the assumption of cracked components leads to over-conservative failure estimations. Among the different alternative approaches proposed that are based on the apparent fracture toughness, $K_{c}^{N}$ is considered. In so doing, the current deterministic underlying concept must be replaced by a probabilistic one to take into account the variability observed in the failure results in order to ensure a reliable design. In this paper, an approach based on the critical distance principle is proposed for the failure assessment of notched EPOLAM 2025 CT samples with each different notch radii (ρ) including a probabilistic assessment of the failure prediction. First, each apparent fracture toughness is transformed into the equivalent fracture toughness for ρ=0 based on the critical distances theory. Then, once all results are normalized to the same basic conditions, a Weibull cumulative distribution function is fitted, allowing the probability of failure to be predicted for different notch radii. In this way, the total number of the specimens tested in the experimental campaign is reduced, whereas the reliability of the material characterization improves. Finally, the applicability of the proposed methodology is illustrated by an example using the own experimental campaign performed on EPOLAM 2025 CT specimens with different notch radii (ρ).

## 1. Introduction

Epoxy resins are one of the most important cross-linked polymers in the family of thermosets [1,2,3]. The outstanding performance of these polymers promotes its use in many mechanical and structural components [4,5], particularly in aerospace and automotive applications. Meanwhile, new application fields are steadily emerging, such as those based on the inclusion of nano- and microparticles to develop toughened epoxy-based materials [6,7,8,9,10]. Two possible failure modes are observed in these materials: those initiated at cracks, and those arising from notches. A number of different representative constructive and geometric details, such as holes, corners, joints, cross section changes, and so forth represent stress concentrations that can be envisaged as notches from a failure behavior viewpoint [11,12,13]. Hence, it is important to predict the failure of these components based on a reliable probabilistic assessment. In particular, failure prediction is of interest in the practical mechanical design of real notched components made of polymers. This stimulates investigation programs to provide the systematic characterization of epoxy resins under fracture and fatigue.

Due to the variety of mechanical behavior exhibited by the different epoxy resin families, specific models have to be applied to ensure a reliable fracture prediction. The markedly brittle behavior of EPOLAM 2025 epoxy resin and the ubiquitous constructive details in which fractures initiate, contemplated as notches, deserve special attention in practical design. Nevertheless, EPOLAM 2025 is a not yet well-known epoxy resin from which apart from technical and commercial specifications, few information is available about the fracture behavior of this material. In any case, the interest of the characterization of the EPOLAM 2025 is justified mainly as a potential candidate to be used as a thermoset matrix of composite materials for aeronautical applications, whereas less emphasis is placed on the simple characterization of this material as a mere terminal adhesive.

Different approaches are applied to design notched elements as cracked components using the apparent or notch fracture toughness $K_{c}^{N}$ as a reference which, in general, leads to failure predictions that are too conservative [14,15,16,17,18,19]. Besides this, these approaches are based on a deterministic concept disregarding the high variability observed in the failure results, so that a probabilistic concept must be integrated in the proposed models if a reliable and suitable design of real components is intended.

In this work, the theory of critical distances is used for the failure assessment of notched EPOLAM 2025 CT specimens with the same specimen thickness, but different notch radii (ρ). This implies the previous calculation of the apparent fracture toughness, then its subsequent transformation into the equivalent fracture toughness for the notch radius ρ=0, and, finally, fitting of a Weibull cumulative distribution function once all the results are reduced to the same basic conditions, allowing the probability of failure to be predicted for different notch radii. In this way, the total number of the specimens tested in the experimental campaign is reduced, whereas the reliability of the material characterization is improved because of the variety of notch geometries and specimens implied in the assessment. Finally, the applicability of the proposed methodology is illustrated with a practical application using the experimental campaign performed on EPOLAM 2025 CT specimens with different notch radii (ρ) performed by the authors.

The normalization proposed herein contributes to an advance in the resolution of the constraint problem in notched elements, since different notch radii can be contemplated as different constraint condition cases even if the same specimen thickness is kept constant within the different samples.

The paper is organized as follows. After this Introduction, in Section 2 the mechanical characteristics of the material used, EPOLAM 2025 epoxy resin, are presented together with the experimental method to measure the fracture toughness. In Section 3, the proposed methodology is introduced in detail. In Section 4, the experimental fracture results are used to illustrate the applicability of the proposed methodology. In Section 5 a discussion about the interpretation of the experimental results is presented and, finally, the main conclusions drawn from this work are given in Section 6.

## 2. Materials and Methods

### 2.1. Material

The material under study was an epoxy resin denoted as EPOLAM 2025. The raw resin and hardener was supplied by AXSON Technologies [20] (Barcelona, Spain), while the posterior mixing and curing processes were performed in the National Institute of Aerospace Technology (INTA), Madrid, Spain. Manufacturing of resin plates was performed by casting a two-component liquid appearance system, with EPOLAM 2025 epoxy resin and an EPOLAM 2025 amine hardener, mixed with a weight ratio of 100 parts of epoxy resin and 28 parts of amine hardener. Mixing was performed very slowly in order to avoid air bubbles being trapped into the liquid system as much as possible. Metallic alloy plates covered with Tooltec PTFE film were used as casting moulds where EPOLAM 2025 was carefully poured from a beaker that was used to prepare the correct weight mix ratio by using a precision balance. Before pouring, the resin/hardener mix was held for 5 min in a vacuum oven for outgassing or elimination of trapped air in the mix. Hardening took place within 24 h at room temperature. Postcuring was performed by means of a computer-controlled Scholz autoclave, in accordance with the following schedule, with a temperature ramp of 30 °C/h: 2 h at 40 °C, 2 h at 60 °C, 2 h at 80 °C, 2 h at 100 °C, and, finally, 2 h at 120 °C. Cooling from this last temperature to room temperature was performed at a maximum rate of 30 °C/h. Moreover, the blend is considered to be stoichiometric so that the chemical reaction is considered to be completed without free remaining functional groups of the resin. For the same reasons, abnormal curing reactions were discarded.

**Figure 1:**
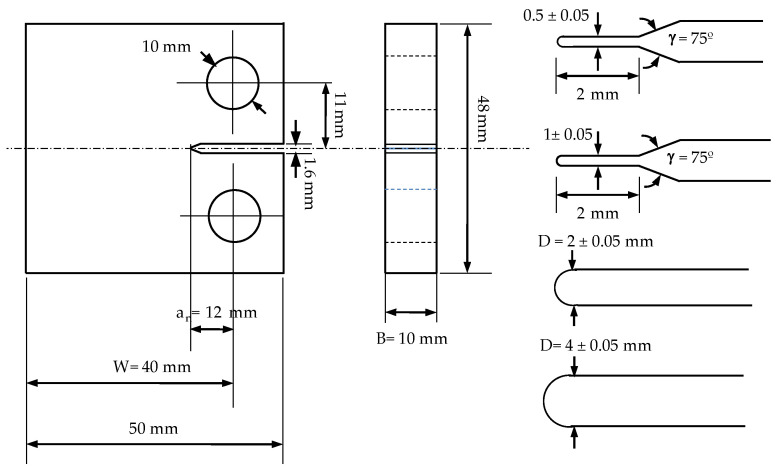
Dimensions of the tested CT specimens with the mechanized notch radii (0.25, 0.5, 1, 2 mm).

The mechanical properties obtained from uniaxial tensile tests are shown in Table 1, with the glass transition of the epoxy resin as 135 °C. The CT samples were machined by PRODINTEC (Gijón, Spain) for four different notch radii according to the geometrical details depicted in Figure 1. The notch radii varies within $\rho =\left\{0.25,0.5,1,2\right\}$ mm, while other dimensions were kept constant. A total of five specimens for each of the notch radii were manufactured, implying a total of 20 manufactured samples.

### 2.2. Experimental Method to Measure Fracture Toughness

The fracture characterization of CT samples was performed according to the ASTM D5045 standard [21], at a strain rate of 0.5 mm/min at ambient temperature in a MTS machine and a load cell of 5 kN. Each fracture test was repeated five times for each of the notch radii.

## 3. The Methodology to Perform the Fracture Assessment of EPOLAM 2025

Global and local methodologies, so denoted in allusion to the way in which the failure criterion is determined, that is, as referred to a driving force representing the hazard of the critical condition or the concatenation of local critical conditions, can be selected to characterize probabilistic fractures of polymeric notched materials [22]. The former is referred to as a unique driving force representative of the hazard of the critical condition being achieved, whereas the latter provides the global probability as a concatenation of local critical conditions. In the present work, only the global approach, based on the theory of critical distances (TCD), is applied for the probabilistic fracture assessment of notched components of EPOLAM 2025, while the local approach is discarded and planned as the subject of future investigations in the modality of the generalized local model (GLM) [23].

**Figure 2:**
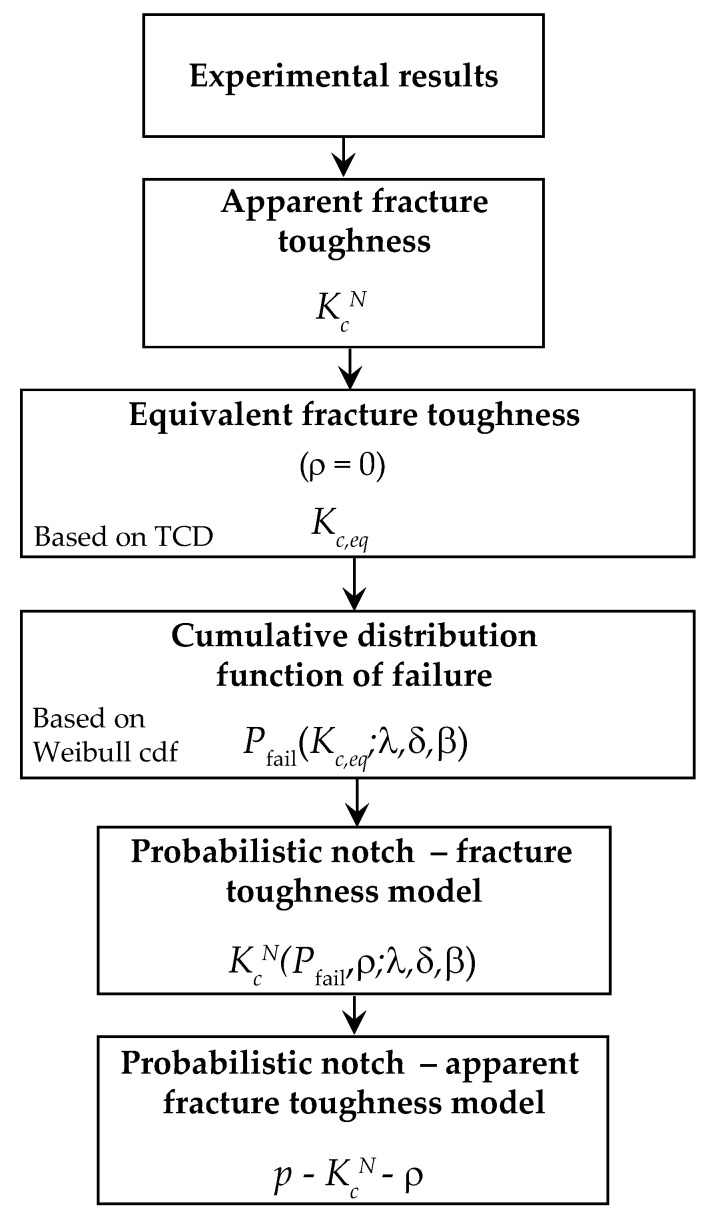
Flowchart of the proposed methodology.

The proposed methodology has satisfactorily been applied to metallic materials in previous works [13,24], that includes different notch radii conditions, that is, the notch effect, as well as different temperatures. In the present research, the fracture experimental campaign was carried out on EPOLAM 2025 at ambient temperature so that the temperature effect was not considered, as illustrated in the flowchart in Figure 2 and described in detail in the following subsections.

### 3.1. Determination of the Apparent Fracture Toughness $K_{c}^{N}$

The application of the cracked specimen formulation to notched specimens according to ASTM D5045-99 [21] allowed the apparent fracture toughness $K_{c}^{N}$ to be obtained from fracture tests in CT samples, that is,

(1) $$K_{c}^{N}=\frac{P}{BW^{1/2}}f\left(x\right),$$

where *P* is the maximum load, *B* the specimen thickness, *W* the specimen width, *a* the crack length, *x* is defined as the ratio x=a/W, and f(x) is defined as follows:

(2) $$f\left(x\right)=\frac{\left(2+x\right)\left(0.8866+4.64x-13.32x^{2}+14.72x^{3}-5.6x^{4}\right)}{{\left(1-x\right)}^{3/2}}.$$

### 3.2. Conversion to the Equivalent Apparent Fracture Toughness $K_{c,eq}$

Once the apparent fracture toughness is obtained, the proposed methodology applies the TCD method [25] to account for the notch radius effect. According to the elemental version of the TCD, that is, the Point Method (PM), fractures will occur when the stress value at a distance L/2 of the notch tip reaches the intrinsic critical strength $\sigma_{0}$, that is,

(3) $$\sigma \left(\frac{L}{2}\right)=\sigma_{0},$$

where the *L* parameter is defined as a characteristic of the material according to the following expression:

(4) $$L=\frac{1}{\pi }{\left(\frac{K_{c}}{\sigma_{0}}\right)}^{2},$$

and $K_{c}$ is the critical stress intensity factor, that is, the fracture toughness of the material. The critical stress value $\sigma_{0}$ must be calibrated, usually being larger than the ultimate strength of the material $\sigma_{u}$.

As a natural extension of the PM, the Line Method (LM) proposes that failure occurs when the average stress over the length 2L over a distance r=0 to 2L, that is:

(5) $$\frac{1}{2L}\int_{0}^{2L}\sigma \left(r\right)dr=\sigma_{0}$$

reaches the critical value $\sigma_{0}$, where *L* represents the same characteristic parameter of the material given by Equation (4).

To describe the notch effect using the TCD, simple classical formulations of the stress field around the notch, as proposed by Creager and Paris [26], can be used [14]:

(6) $$\sigma \left(r\right)=\frac{K}{\sqrt{\pi }}\frac{2(r+\rho )}{{\left(2r+\rho \right)}^{3/2}},$$

which allows the stress field near the notch root to be approximated for notches of length *a* and root radius ρ when ρ<<a, *K* being the stress intensity factor. In fact, by applying the LM approach according to Equation (5), Equation (6) transforms into an easier and equivalent formulation for the apparent fracture toughness:

(7) $$K_{c}^{N}=K_{c}\sqrt{1+\frac{\rho }{4L}},$$

with the critical distance *L* as the model parameter. As a result, the TCD method in the LM version defines the apparent fracture toughness $K_{c}^{N}$ as a function of the fracture toughness of the material $K_{c}$ and a factor depending on both the notch radius ρ and the critical distance parameter *L*.

The proposed methodology suggests inverting the previous Equation (7) to convert experimental values for different notch radii conditions into an equivalent case with ρ=0. In other words, solving Equation (7) for $K_{c}$, the following definition of the equivalent fracture toughness $K_{c,eq}$ results in:

(8) $$K_{c,eq}=K_{c}^{N}\sqrt{\frac{4L}{4L+\rho }}.$$

Accordingly, the equivalent fracture toughness $K_{c,eq}$ is obtained for the reference value ρ=0 by replacing the experimental apparent fracture toughness and the corresponding notch radius, ρ, into Equation (8). In this way, the proposed methodology allows the expeirmental fracture results from different notch radii conditions, that is, $\left(K_{c_{1}}^{N},K_{c_{2}}^{N},\ldots ,K_{c_{n}}^{N}\right)$, to be jointly considered in the fracture characterization of the material by defining the equivalent set of fracture toughness $\left(K_{c,eq_{1}},K_{c,eq_{2}},\ldots ,K_{c,eq_{n}}\right)$ regardless of the notch radius effect.

### 3.3. Derivation of the Cumulative Distribution of Failure $P_{fail}$

In this step, the equivalent fracture toughness values obtained from the conversion in Equation (8) are used to derive the probabilistic approach. Indeed, the probability of failure $P_{\mathrm{fail}}$ can be defined associated to each *i*-th equivalent fracture toughness based on the application of Bernard’s plotting position formula [27,28,29],

(9) $$P_{{\mathrm{fail}}_{i}}=\frac{i-0.3}{n+0.4},\;i=1,2,...,n,$$

where *n* represents the total number of experimental results.

Then, according to the minimal conditions related to the fracture criterion [28,29], a minimal three-parametric Weibull distribution is proposed to probabilistically define the equivalent fracture toughness $K_{c,eq}$, that is,

(10) $$P_{\mathrm{fail}}\left(K_{c,eq};\lambda ,\delta ,\beta \right)=1-\exp\left[-{\left(\frac{K_{c,eq}-\lambda }{\delta }\right)}^{\beta }\right],\;\;K_{c,eq}>\lambda ,$$

where λ,δ,β are the location, scale, and shape parameters of Weibull distribution, respectively. Note that all experimental results obtained for the different notch radii are jointly evaluated as a unique cumulative distribution function (cdf), thus ensuring higher reliability thanks to the proposed methodology.

### 3.4. Derivation of the Probabilistic $K_{c}^{N}$ vs. $\rho^{0.5}$ Field

Finally, the probabilistic definition of the equivalent fracture toughness for ρ=0 is now used to derive the iso-probability percentile curves for any other notch radii by applying the TCD method again, that is, by combining Equation (8) and Equation (10):

(11) $$K_{c}^{N}\left(\rho ,P_{\mathrm{fail}};\lambda ,\delta ,\beta \right)=\left[\lambda +\delta {\left[-\log(1-P_{\mathrm{fail}})\right]}^{1/\beta }\right]\sqrt{1+\frac{\rho }{4L}},$$

with λ,δ,β and *L* as parameters.

**Figure 3:**
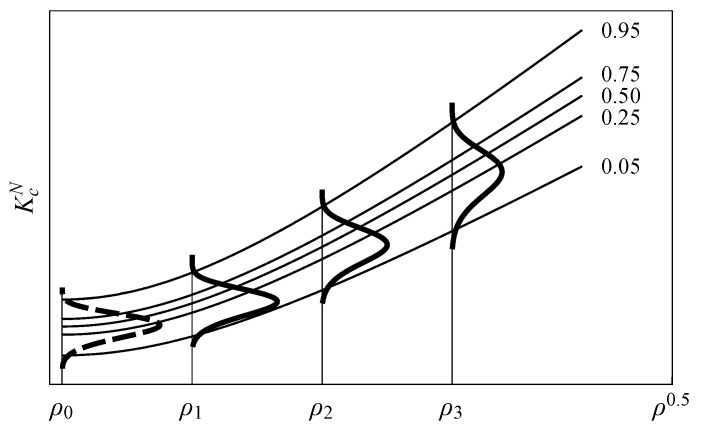
Schematic illustration of the probabilistic $K_{c}^{N}-\rho^{0.5}$ field for the proposed model.

Figure 3 illustrates the resulting probabilistic *p*-percentile curves of the $K_{c}^{N}-\rho$ field. Note the robustness of the proposal compared with deterministic approaches, since the inherent variability typically observed in fracture tests is now included in the failure prediction for any notch radii condition at the given *p*-percentile of interest, providing more reliable material information for the practical design.

## 4. Results

In this section, the results from the fracture characterization of EPOLAM 2025 are firstly presented and, subsequently, evaluated as a practical example to illustrate the applicability of the proposed methodology.

### 4.1. Fracture Results

**Figure 4:**
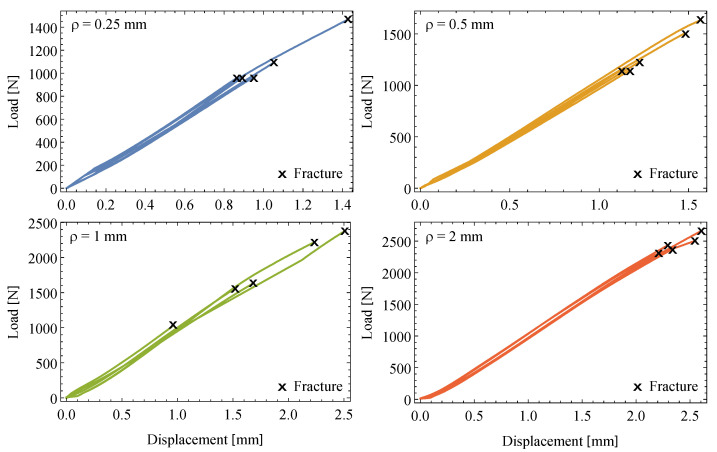
Experimental load-displacement plots for the different samples of EPOLAM 2025 with notch radii, ρ=0.25,05,1 and 2 mm.

The load-displacement curves from the different samples, each of them consisting of specimens with distinct notch radius, illustrate the trend of increasing failure loads for increasing notch radii, as shown in Figure 4 along with the predominant linear elastic behavior until failure. The linear trends exhibit nearly the same slope value for different notch radii. Practical coincidence is confirmed among the slope values of the load-displacement curves for all the specimens pertaining to the different samples.

**Figure 5:**
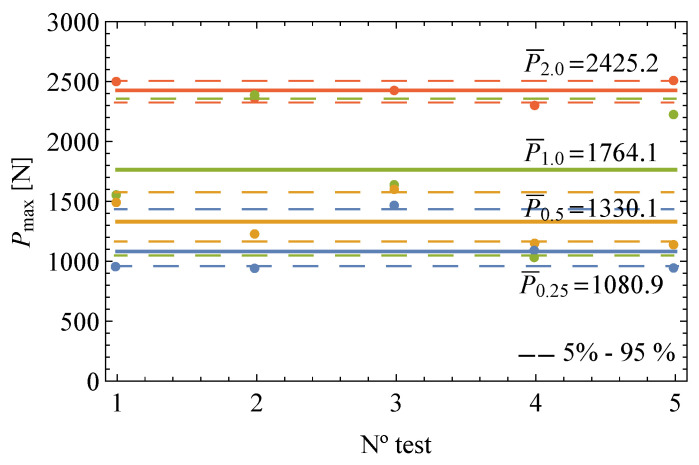
Experimental maximum load values obtained for the different notch radii samples of EPOLAM 2025 with the standard deviations indicated.

Figure 5 depicts the maximum load values reached for each of the different notch radii. Contrary to the narrow scatter observed among the slopes of the near-linear load-displacement plots, a large variability among the results of the maximum load is noticed for the different samples showing the same notch radius, particularly for lower values. Table 2 gathers the experimental results for all samples with the corresponding apparent fracture toughness according to Equation (1) and the mean and standard deviation values along with the geometric details of the specimens.

### 4.2. Fracture Assessment

The suitability of the proposed methodology is illustrated with its application to the experimental campaign on EPOLAM 2025 encompassing those steps previously described in Section 3 and graphically described in the flowchart of Figure 2.

**Figure 6:**
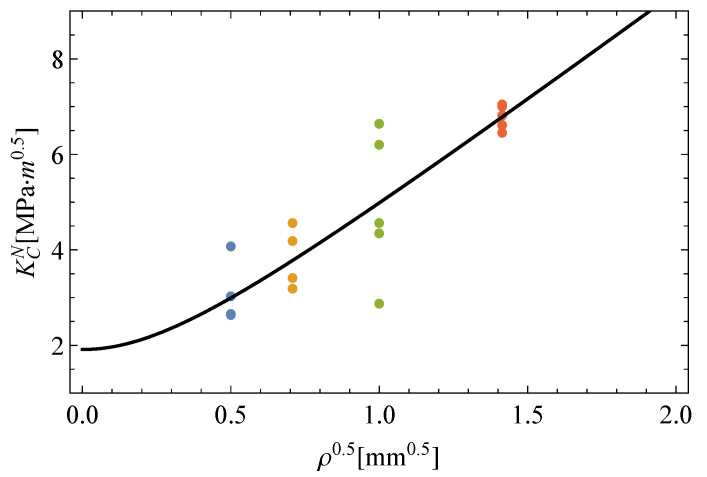
Experimental results obtained for notch fracture toughness and the estimation according to the TCD method.

**Figure 7:**
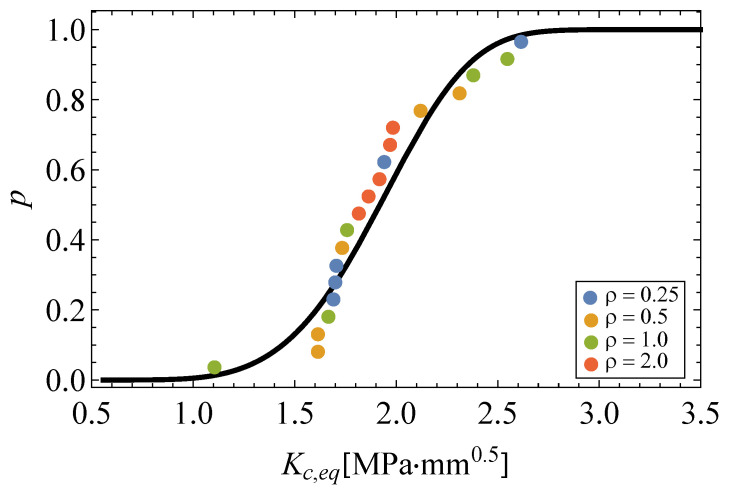
Equivalent fracture toughness and Weibull distribution based on the Bernard plotting position.

**Figure 8:**
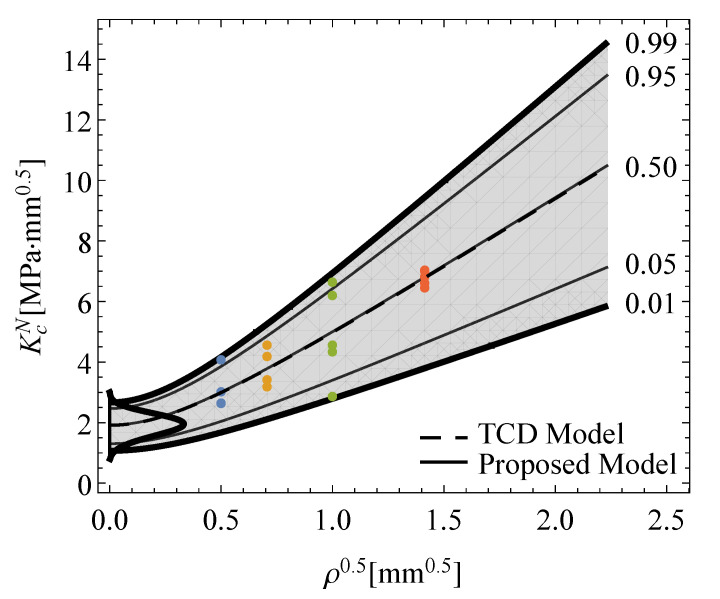
Theoretical predictions of the probabilistic $K_{c}^{N}-\rho^{0.5}$ field and the experimental values for the apparent fracture toughness of EPOLAM 2025.

− ***Step 1: Estimation of the TCD method***. Herewith, the critical distance *L* is calculated according to the TCD method and the fracture toughness of the material $K_{c}$ by fitting Equation (7) to the experimental apparent fracture toughness based on the Least Squares (LS) method. The following estimates are given:
  (12) $$K_{c}=1.913;\;L/2=0.0432.$$
  Figure 6 illustrates the predicted $K_{c}^{N}-\rho^{0.5}$ curve resulting from the TCD method to describe the notch effect in EPOLAM 2025 along with the experimental values. In this way, a reasonable estimation of the apparent fracture toughness is confirmed with the use of the TCD method [30,31]. Two zones are characterized: that for lower values in which the notch radius effect is nearly negligible, and another one in which this effect becomes significant from a certain point. Though the inherent variability of the fracture is noticeable, its prediction is not possible using this deterministic approach.
− ***Step 2: Conversion to the equivalent fracture toughness*$K_{c,eq}$**. Based on the TCD method, the original *i*-th value of the apparent fracture toughness $K_{c_{i}}^{N}$ for the *j*-th notch radius can be transformed to the equivalent fracture toughness for ρ=0, according to Equation (8), as follows:
  (13) $$K_{c,eq_{ij}}=K_{c_{i}}^{N}\sqrt{\frac{4L}{4L+\rho_{j}}},\;\;i=1,2,\ldots ,n;\;\;j=1,2,\ldots m$$
− ***Step 3: Derivation of the cumulative distribution of failure*$P_{\mathrm{fail}}$**. Once the equivalent fracture toughness values have been obtained from experimental results, they can be considered as random samples following a Weibull distribution according to Equation (10). Thus, by ordering this set and applying a plotting position scheme, the experimental cumulative distribution function of the equivalent fracture toughness is obtained and consequently fitted, providing the following estimates for the Weibull parameters:
  (14) $$P_{\mathrm{fail}}=1-\exp\left[-{\left(\frac{K_{c,eq}-0.556}{1.483}\right)}^{4.344}\right].$$
  Figure 7 depicts the resulting equivalent fracture toughness values and the estimated Weibull cdf. It is worth mentioning that all fracture results from the experimental campaign, coming from different notch radii conditions, can now be pooled into a single cdf and jointly evaluated as such.
− ***Step 4: Derivation of the probabilistic*$K_{c}^{N}-\rho^{0.5}$*field***. Finally, the probabilistic definition of the equivalent fracture toughness can now be extended to any other notch radii condition based on the TCD method by substituting the equivalent fracture toughness $K_{c}^{N}$ from Equation (14) in Equation (13), resulting in the percentile curves in the $K_{c}^{N}-\rho^{0.5}$ field:
  (15) $$K_{c}^{N}\left(\rho ;P_{\mathrm{fail}}\right)=\left[0.556+1.483{\left[-\log(1-P_{\mathrm{fail}})\right]}^{0.230}\right]\sqrt{1+\frac{\rho }{0.1728}},$$
  as a function of the notch radius for a given $P_{\mathrm{fail}}$ of interest.
  Figure 8 shows the predictions of the proposed methodology with the experimental campaign of EPOLAM 2025 for different notch radii, besides the corresponding pdf of the equivalent fracture toughness from Figure 7, where all experimental values can be pooled together. As can be seen, the experimental fracture results can be satisfactorily predicted including the non-negligible scatter, being randomly distributed around the percentiles for each given notch radius. Note also that the median percentile (p=0.5) is coincident with the corresponding curve from the TCD method, but the proposed methodology allows any other percentile curve of interest to be derived, thus enhancing the deterministic approaches by predicting the inherent variability in fracture epoxi resin results for any notch radii condition.

**Figure 9:**
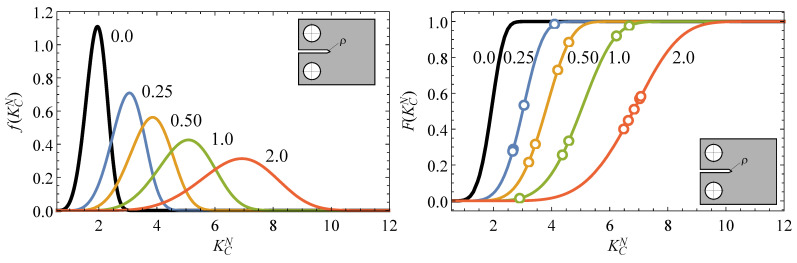
Theoretical predictions of the pdf and cdf of the apparent fracture toughness for different notch radii conditions and the experimental values obtained from EPOLAM 2025.

As an additional and direct result from the proposed methodology, Equation (15) also allows the probability distribution function (pdf) and cumulative distribution function (cdf) of the apparent fracture toughness, that is, $f(K_{c}^{N})$ and $F(K_{c}^{N})$, to be derived for any notch radius condition, as can be seen in Figure 9, where the experimental results are also plotted in the cdfs for each of the notch radii.

### 4.3. Numerical Simulations

**Figure 10:**
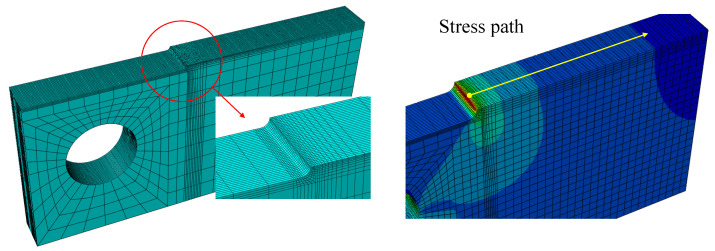
Finite element model for one-half of the CT symmetric specimen used in the numerical simulations (**left**) and the geometrical path selected (**right**).

Although the proposed methodology allows the critical distance parameter *L* to be estimated, as shown in previous steps, a numerical simulation has been conducted on CT EPOLAM specimens to compute the stress-distance curves along paths close to the notch root radius in order to corroborate this estimation, as prescribed in the TCD method. Figure 10 illustrates the finite element model of half the CT specimen used in the numerical simulations and the geometrical path over which the evaluation has been performed for each of the different notch radii. The numerical model was assembled in ABAQUS CAE using quadratic 3D hexahedral elements (C3D20R). The EPOLAM was modelled as a linear elastic material using the properties presented in Table 1. For each type of CT specimen, the average maximum force obtained in experiments was used as loading conditions in the FE simulations.

**Figure 11:**
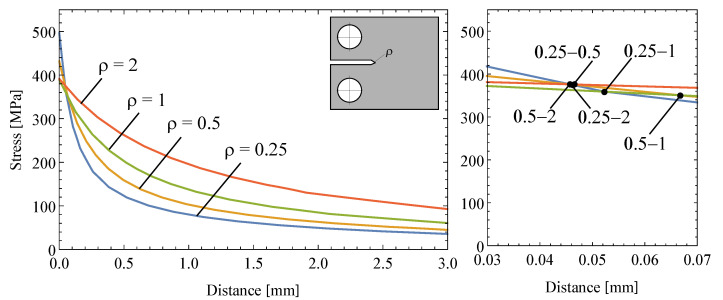
Stress-distance curves of the path obtained from numerical simulations for different notch radii conditions (**left**) and multiple intersections among these curves close to the notch root radius (**right**).

From the numerical results, the stress-distance curves for each of the different notch radii were obtained (see Figure 11 (left)). Different intersection points can be identified in a zoomed view for distance values lower than 0.07 mm, as shown in Figure 11 (right), justifying the selection of the Line Method instead of the Point Method. Taking the mean value of these intersection points as a reference allows the critical distance parameter *L* obtained from the proposed methodology in Equation (12) to be satisfactorily corroborated.

## 5. Discussion

It is apparent that though the specimen thickness is maintained constant in all the specimens, the constraint conditions, that is, the triaxiality, varies just as a function of the different notch radii so that the procedure could be extended to any joint evaluation implying different thicknesses. Nevertheless, to study the general case of constraint, a local model is recommended as a seemingly more reliable methodology [32].

The joint assessment based on pooling all the results from the different samples related to either notch radius has two advantages. On the one side, an increasing number of notch radii are involved in the experimental campaign for a given total number of tests. This ensures a more representative assessment of the influence of the geometry diversity. On the other side, the suitability of the model is simultaneously checked for different notch radii and the model parameters are estimated on a more robust basis.

Regarding the possible brittle or ductile behaviour of the polymer used in this study, some comments should be emphasized. On the one hand, the load-displacement curves shown in Figure 4 seem to indicate that there is no ductility at the scale related to the experimental campaign performed, and the behaviour is considered fully brittle. Furthermore, the high repetitively of the slopes suggest negligible scatter associated to the Young Modulus of the material. For those reasons, the authors considered to adopt a brittle approach, including linear elastic finite element models and fracture criterium (apparent fracture toughness). On the other hand, it is true that the ductility associated to polymers could play a role near the notch types. Nevertheless, the consideration of the TCD to predict the fracture toughness of the material minimizes the effect of local plasticity, because the critical stress associated to the TCD ($\sigma_{0}$) is measured at a certain critical distance ahead of the notch (L/2), and not just in the notch type, where the ductile behaviour could be higher. In any case, the authors want to remark that further investigations about the application of the methodology proposed in this paper by including the local ductile behaviour of the material could slightly improve the results.

## 6. Conclusions

A methodology based on the theory of critical distance has been derived for probabilistic prediction of the apparent fracture toughness of notches for any notch radii condition. This proposed methodology allows all experimental results for different notch radii conditions to be pooled together and evaluated as a single Weibull cdf distribution. Furthermore, a numerical simulation has been conducted on CT specimens to corroborate the estimation procedure of the critical distance parameter *L* proposed in the probabilistic methodology, based on the stress-distance curves over the path close to the notch root radius. Finally, the applicability of the proposed methodology has been demonstrated by its application to an experimental campaign conducted on EPOLAM 2025 for four different notch radii with a total of 20 samples.

## Figures and Tables

**Table 1:** EPOLAM 2025 tensile properties.

*E* [GPa]	ν
3.2	0.36

**Table 2:** Results from the experimental program on CT specimens of EPOLAM 2025.

Set	Specimen	*W*	*B*	ρ	Max. Load	$K_{c}^{N}$	M(SD)
		[mm]	[mm]	[mm]	[N]	[MPa mm${}^{1/2}$]	[MPa mm${}^{1/2}$]
0.25	0.25-1	40	10	0.25	956.33	2.68	3.03 (0.62)
	0.25-2				950.22	2.67	
	0.25-3				1462.07	4.10	
	0.25-4				1088.78	3.05	
	0.25-5				947.38	2.66	
0.5	0.5-1	40	10	0.5	1500.02	4.21	3.73 (0.64)
	0.5-2				1226.58	3.44	
	0.5-3				1634.67	4.59	
	0.5-4				1144.39	3.21	
	0.5-5				1145.23	3.21	
1.0	1.0-1	40	10	1.0	1554.92	4.37	4.95 (1.55)
	1.0-2				2376.10	6.67	
	1.0-3				1635.97	4.59	
	1.0-4				1033.53	2.90	
	1.0-5				2220.01	6.23	
2.0	2.0-1	40	10	2.0	2504.68	7.03	6.81 (0.25)
	2.0-2				2364.46	6.64	
	2.0-3				2434.76	6.84	
	2.0-4				2307.10	6.48	
	2.0-5				2517.61	7.07	
